# Snack Development for School Feeding Programs in Africa: A Scoping Review

**DOI:** 10.3390/ijerph17144967

**Published:** 2020-07-10

**Authors:** Saffia Hassanally, Ashika Naicker, Evonne Singh

**Affiliations:** Department of Applied Sciences, Consumer Sciences: Food and Nutrition, Durban University of Technology, 70 Steve Biko Road, Musgrave, Berea 4001, South Africa; ashikan@dut.ac.za (A.N.); evonnes@dut.ac.za (E.S.)

**Keywords:** school feeding program, snacks, healthy, learners, primary school, energy-dense

## Abstract

The benefits of school feeding have been well documented. As such, school feeding programs have continuously gained popularity in developing countries. However, challenges and potential opportunities persist, calling for a review of school feeding for long-term sustainability. South Africa has an opportunity to improve their National School Nutrition Program (NSNP) by including an energy-dense snack that would increase the recommended dietary allowance (RDA) of school children to meet at least 25% of their energy requirements. The objective of this scoping review was to conduct a review and an appraisal of studies on snack food development for school feeding programs in Africa. Eligible studies had to report snack development for school feeding programs in Africa. We conducted an electronic search in National Research Foundation (NRF) NEXUS, Elton B. Stephens Company (EBSCO), International Food Information Service (IFIS), Nutrition and Food Sciences Center for Agriculture and Bioscience International (CABI.ORG), and Google Scholar. Of the 429 articles identified, nine studies were included in the final review—five from within South Africa and four from elsewhere in Africa. Data extracted included the study design, intervention, outcomes, relevant findings, and limitations. Results were presented in a narrative summary. The review findings showed that energy-rich staple foods and food fortification were commonly used in snack development. The popular snack products developed included porridges and biscuits. While most studies reported nutritional outcomes, few studies reported on sensory acceptability tests and only two studies conducted a cost analysis. This review of previous snack development initiatives for school feeding programs in Africa underscores the importance of establishing the sustainability of any food product developed. The findings of this review have the potential to inform future snack product development for school feeding programs.

## 1. Introduction

It has become increasingly important to many heads of state, governments, and organizations around the world to end world hunger and improve food security for all regions in the world [1]. School feeding has been identified as a means of addressing the Millennium Development Goals 1 and 2, which aim to halve the proportion of people suffering from hunger worldwide and to ensure universal enrolment in primary school education [2,3]. Several government policies have been initiated to address the issue of food insecurity in South Africa, such as the Food Fortification Program, food supplementation, and school feeding programs, including the National School Nutrition Program (NSNP), as well as daycare center schemes [4]. In 1994, a national scale primary school feeding program was established by the South African Department of Health and was taken over by the Department of Education in 2004 [3]. The latest figures from the 2019/20 Basic Education Department Budget Vote speech indicate that 1.6 billion ZAR (89736464 USD) was allocated to the NSNP in South Africa [5]. The NSNP reaches approximately 20,000 schools that are classified among the three poorest quintiles and provides meals to more than nine million learners nationwide [6]. The NSNP aims to enhance the learning capacity of learners by providing a healthy school lunch meal to increase learners’ energy levels, as well as improving micronutrient intake, making them alert and receptive during lessons [7]. Currently, the South African School Feeding Program provides only 18% of the recommended dietary allowance (RDA) for children [8,9]. South Africa has an opportunity to improve current gaps in its school feeding program. The recent report on the National School Nutrition Program in South Africa recommended the development of an energy-dense snack to increase the recommended dietary allowance (RDA) of school children to meet at least 25–30% of their energy requirements, which will also address other critical issues, such as micronutrient deficiencies and hunger [9]. The three major micronutrients that almost one-third of the world’s population lack are iodine, iron, and vitamin A, deficiencies of which can result in severe consequences, including learning disabilities in children, impaired work capacity, serious illness, and death [10,11]. Micronutrient deficiencies are common among school children, but unlike stunting and various other effects of long-term malnutrition, micronutrient deficiencies are reversible with the use food intervention strategies, such as school fortification feeding programs or the addition of supplements into school feeding programs [3]. School feeding programs not only reduce short term hunger but alleviate the effects of malnutrition on learning capacity, having a greater impact on the improvement of learner performance in tests and exams [6]. In addition to providing an incentive to attend school, implementation of a school feeding program may also reduce absenteeism. A review of studies showed that school feeding was associated with an estimated average of four to six additional days of attendance at school per child per year, which could be due to the attraction of a free meal [12,13,14]. The benefits of snacks in the school feeding programs are well established. Snacks alleviate short-term hunger and micronutrient deficiencies, improve learning, and are cheaper and easier to distribute than meals [2]. In accordance to business plans and minimum feeding requirements as outlined by the South African National Treasury department, the suitable average cost per meal per learner per day (inclusive of cooking fuel and honorarium) should be 2.85 ZAR (0.16 USD) for primary schools and 3.60 ZAR (0.20 USD) for secondary schools in South Africa; hence, the inclusion of a snack has to be cost-effective [9].

The food product development process from concept to commercialization plays a crucial role in the success of new food products; however, some products have a high failure rate due to low investment rates in research and development activities and lack of incorporation of consumer preferences in the product development process [15,16]. Research in snack development for school feeding programs in Africa has mainly been driven by food security, health, or educational outcomes; however, it is not clear how the product development process is integrated and what impact it has for country-wide scalability.

Scoping reviews provide an overview and analysis of the available research evidence without providing a summary answer to a discrete research question [17]. This paper aims to identify and review the literature of the snack product development for school feeding programs that have been developed in Africa to inform future snack development for school feeding programs.

## 2. Materials and Methods

A scoping review was conducted to synthesize research evidence on snack product interventions developed for school feeding programs in Africa. In reporting this review, we have been guided by the Preferred Reporting Items for Systematic Reviews and Meta-Analyses (PRISMA) extension for Scoping Reviews (PRISMA-ScR) guidelines [18]. Our search strategy was formulated based on knowledge of the literature, using the following keywords to broaden the retrieval of relevant articles: “energy-dense”, “snack foods”, “cost-effective”, “developing countries”, “healthy snacks” “primary school children”, “Africa”, “nutrition feeding program”. Five databases were reviewed for articles, including National Research Foundation (NRF) NEXUS, Elton B. Stephens Company (EBSCO), International Food Information Service (IFIS), Nutrition and Food Sciences Center for Agriculture and Bioscience International (CABI.ORG), and Google Scholar. We imported all papers (titles and abstracts) into an endnote database and removed duplicates. Using the pre-specified inclusion criteria, the article’s titles and abstracts were screened by one independent reviewer. Relevant information on the study design, duration, number of participants, age of study population, the type of product developed, outcomes, results, and limitations were extracted. The critical appraisal of studies included the adherence to the steps of food product development. Other considerations included in the appraisal were packaging, storage, transport and food safety. Conformance to the product development was assessed using the model by Fuller, as follows: (1) idea generation, where the company looks at the consumers’ needs and forms an idea, involving technical marketing and manufacturing personnel; (2) screening of ideas, where the company looks at the feasibility of the idea and whether it is marketable; (3) development, where a trial product is produced and goes through a multitude of tests, including objective testing, consumer preference testing, market testing, and evaluation; (4) production, involving the establishment of certain requirements and specifications of the product; (5) consumer trials, where the product goes through a series of trials that determine the consumer acceptability of the product using the skills of trained sensory analysis panelists; (6) test marketing, where the product is released onto the market and is observed over a period of time. Fuller’s theory is more acceptable for developing food products as it makes use of subjective methods to measure the sensory properties of new food products, which are believed to be more accurate in determining consumer acceptability and preference [19,20]. Studies reporting on snack development for school feeding programs in Africa for both primary and secondary school children were eligible for the review. Articles published in English and unpublished dissertations and theses for the period 2000–2020 were included in the review. The included studies were not limited to reporting snack product development, but also included studies reporting health and educational outcomes from the snack product development. The age inclusion criteria covered the general school age of children in Africa, which was guided by the South African Schools Act of 1996, whereby children aged 7–15 are compelled to attend school; and the RDA categories for children: 4–8 years, 9–13 years, and 14–18 years [21,22]. The study designs included experimental, randomized, and non-randomized control trials; cross-sectional designs; and pre- and post-test designs. Data were extracted by two reviewers (S.H., A.N.) into a master table. All extractions were checked for accuracy by a third reviewer (E.S.). Any disagreements were discussed until agreement was reached.

## 3. Results

### 3.1. Study Characteristics

**Figure 1:**
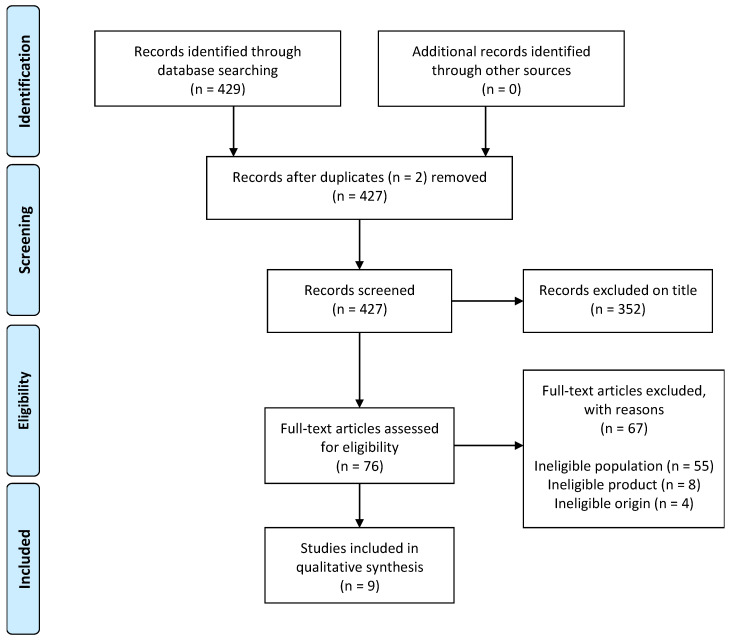
Preferred Reporting Items for Systematic Reviews and Meta-Analyses (PRISMA) flow diagram [23].

Of the 429 unique articles identified, 427 were screened after excluding 2 duplicates. Out of the remaining articles, 352 were excluded because their abstracts and titles did not meet the eligibility requirements (Figure 1). From a full-text review of the remaining 76 articles, a total of 9 articles were identified for the final review. The reasons for excluding 67 studies were that they had ineligible population, products, or origin. The nine studies included in the final review included four from Africa (including two from Tanzania and one each from Kenya and Ethiopia) and five from South Africa.

### 3.2. Review of Previous Snack Developmental Studies

Table 1 includes summaries of articles by study design, product development intervention, outcomes, results, appraisals, and limitations.

### 3.3. Summary of Table 1

From the nine studies reviewed, three were randomized control trials, two were non-randomized controls trails, two were cross-sectional studies, one was an experimental product development study, one was a mixed methods design, and one was a pre- and post-test design. The duration of the studies ranged between 1 month and 2.5 years. Children from rural schools were used for seven of the studies, while the remaining two studies recruited children from urban schools. The types of snacks developed ranged from porridges to biscuits, while one study developed a beverage snack. Five studies reported only nutritional outcomes [24,25,26,27,28]; two studies reported nutritional, sensory, and cost-effectiveness outcomes [29,30]; one study reported nutritional and educational outcomes [31]; and one study reported on only the sensory outcome [32]. For studies conducting anthropometric measurements, standardized methods were used for two out of three studies, using World Health Organization (WHO) growth reference charts appropriate to each study population [28,31]. The remaining study, which conducted anthropometric measurements, did not indicate which methods and growth reference charts were used [24]. Standardized serum retinol measurement methods were used for studies that measured serum retinol levels [24,25,27].

### 3.4. Review of Studies

Studies reviewed assessed modalities used in the product development of snack foods for school feeding programs.

#### 3.4.1. Ingredient Specification

Five out the nine studies used staple foods in the product development of a snack for the school feeding program. In the study by Zivkovich, an uji snack was developed by boiling a mixture of water, ground corn flour, and sugar to create a corn-based porridge [28]. In Kenya, Murphy, Gewa [26] developed three snacks using a local staple food, githeri, as the main ingredient. Three equicaloric snacks were developed: a vegetarian snack, designated the “energy snack” a snack that included beef; and a snack that included whole milk. The energy snack consisted of 230 g of vegetarian githeri, while the meat snack contained 225 g of githeri with 38% cooked minced beef. To include milk in a snack, the amount of vegetarian githeri was reduced to 100 g and a glass of milk (250 g) was included in the snack. Belayneh, Yetneberk [32] developed a porridge, named kinchie, made from quality protein maize (QPM) coarsely milled maize gain, where QPM was compared with conventional maize for school feeding in Ethiopia. In the study by Hochfeld, Graham [31], a fortified cooked porridge with oats, maize, wheat, and sorghum was developed in Johannesburg, South Africa. Lastly, in the study by Kearney [30], a vetkoek was developed, a traditional South African snack similar to a bread-type cake that is fried in oil, measuring 120 g per portion. The ingredients formed part of the most commonly purchased household food items.

Four out of the nine studies used fortified ingredients to develop snacks for the school feeding program. In the first study, a multiple-micronutrient fortified beverage powder with ten micronutrients was developed by food technologists at Procter and Gamble for primary school children in Tanzania [24]. In the second study, a long-term study done in South Africa by van Stuijvenberg, Dhansay [27], a micronutrient-fortified biscuit was developed and served with a vitamin C-fortified cold drink to evaluate the impact of on micronutrient deficiencies in primary school children over 2.5 years. In the third study, Hochfeld, Graham [31] developed a cooked porridge that was fortified for an in-school breakfast program in South Africa. In Kenya, Murphy, Gewa [26] developed three snacks using a local staple food—githeri, a stew of maize, beans, and vegetables—as the main ingredient to address micronutrient deficiencies in rural school children. Three equicaloric snacks were developed primarily from fortified cooking fat. In the remaining studies, orange-fleshed sweet potato was used to assess the effects of β-carotene on the vitamin A status of primary school children [25] and a biscuit enhanced with soy flour was developed to prevent obesity in children in South Africa [29].

#### 3.4.2. Nutritional Outcomes

Seven out of nine studies evaluated the nutritional outcomes of the developed product. The first study evaluated the impact of a 130 kcal supplemental snack (corn-based porridge) on the growth of primary school children living in rural Tanzania over 15 months. Findings showed that there was a significant decline in the mean height-for-age z-scores and mean weight-for-age z-scores from baseline to follow-up [28]. The second study involved the development of githeri, a stew made of local ingredients (maize, beans, and vegetables), to reduce micronutrient deficiencies in rural school children in Kenya. After a two year duration, the effects of snack design on micronutrient deficiencies in rural Kenyan school children were measured. There was a significant increase in energy intake and vitamin B12 by the children who consumed the animal-based snack. Children in the control group increased energy intakes by 18 kJ/d, whereas those in the meat group increased energy intakes by 536 kJ/d [26]. In the third study by Ash et al. [24], a fortified beverage was developed to determine the effect of the micronutrient-fortified beverage on iron status, anemia, vitamin A status, and growth of schoolchildren in Tanzania aged 6–11 years. Results noted non-significant differences at baseline between children in the fortified and non-fortified groups in terms of iron and serum retinol levels; however, at the 6 month follow-up mean incremental changes in weight, height, and body mass index (BMI) were significantly higher in the fortified group than in the non-fortified group. The results also showed that the fortified beverage lowered the overall prevalence of anemia and vitamin A deficiency [24]. Specific nutrient information on fiber was not mentioned in any of the studies.

In the study by Kearney et al. [30], the developed snack contributed 21.6% of energy, 14.4% of calcium, 141% of iron, 62.4% of zinc, and 17.7% of vitamin A for the group of children. In the study by Hochfeld et al., the effects of an in-school breakfast program on the anthropometric and school performance of school children were evaluated. This study showed positive results, as there was a statistically significant nutritional change over the period of the program. A 4.7% decrease in severe stunting levels and an overall 4.3% positive change in the number of children in the category of normal height-for-age limits were seen. Baseline measurements for overweight learners indicated 27.6% of learners were either overweight (1.9%) or severely overweight (10.7%), while follow-up results indicated that the overweight percentage reduced to 13.8% and severely overweight reduced to 6.4%. Learners, educators, and principles indicated that they perceived that the breakfast program had a positive impact on the children’s ability to learn by improving their participation and concentration in the classroom [31].

In another study, primary school learners were fed 125 g of sweet potato for five days during the week as a snack to assess the effect on the vitamin A status of the children. The treatment group were fed orange-fleshed sweet potato, which is rich in β-carotene, while the control group were fed white-fleshed sweet potato. The results of this study showed an improvement in vitamin A stores in the treatment group as compared to the control group, as the proportion of children with low serum retinol concentration (<0.070 µmol/L) after the intervention decreased from 71% to 50% (*p* = 0.001) in the treatment group and decreased from 73% to 49% (*p* = 0.001) in the control group [25]. In the remaining study, the effects of a micronutrient-fortified biscuit on micronutrient deficiencies in primary school children were evaluated. The results indicated that the micronutrient-fortified biscuit was enough to maintain serum retinol concentrations on a day-to-day basis; however, it was not enough to sustain levels during the long school holiday. Urinary iodine levels improved from baseline to follow-up, where the prevalence of low urinary iodine dropped from 97.1% before the intervention to 4.8% after the first 12 months of intervention [27].

#### 3.4.3. Sensory Analysis

Four out of the nine studies conducted sensory analysis. In the study by Murphy et al. [26], sensory analysis was conducted first on study staff and then on children for acceptability; however, the types of sensory tests conducted were not indicated. In the study by Belayneh et al. [32], the results of the sensory analysis showed a strong preference towards the QPM, with 58% of the participants liking QPM kinchie very much, whereas only 1.7% rated conventional maize kinchie in the same category. The overall evaluation showed that approximately 61% of the respondents perceived QPM kinchie as very good, whereas only 5% of the respondents perceived conventional maize as very good. Furthermore, 66% of respondents rated QPM kinchie as having a good texture and 21% rated the texture as very good, whereas 58% of respondents reported conventional maize kinchie as having fair overall sensory characteristics. None of the participants scored QPM kinchie as poor in terms of taste, however 0.8% of participants scored conventional maize kinchie as poor for taste. The sensory acceptability test results for the product developed by Kearney et al. showed that 90% of children found the product to be acceptable and 65% liked the vetkoek very much [30]. In the remaining study by du Plessis [29], sensory evaluation revealed that approval rates for the biscuit were as follows: 58.3% liked the taste, 57% liked the texture, 54.3% liked the color, 59.5% liked the smell, and 48.9% liked the portion size.

#### 3.4.4. Cost of the Developed Product

Two out of the nine studies included the cost factor of the developed product. In the study by du Plessis [29], the cost per 30 g portion of biscuit was priced at R0.55, while in the study by Kearney [30] the vetkoek was priced at R1.50 per portion of 120 g.

### 3.5. Study Appraisal

The reviewed studies were appraised by assessing the conformance to the food product development steps. Other considerations included in the appraisal were packaging, storage, transport, and food safety. The limitations of the studies included a lack of a systematic processes in terms of ideation and screening of ideas and prototype creation, while only four studies conducted consumer acceptance tests (sensory analysis). Only one study indicated the packaging type, while in six studies the snacks were cooked and served immediately on the premises. Three out of the nine studies reported a food safety aspect; one study used microbiological tests, one study indicated that the snack preparation and serving were observed by food monitors, and the remaining study indicated that food handlers received training on food safety and preparation of the snacks.

## 4. Discussion

To find a solution to the impending nutrition insecurity and issues of hunger around the world, further research needs to be conducted. Three possible research outlooks are: (1) Health aspect: Having access to nutritious and safe food is important for individuals to lead a healthy, well-balanced life, therefore research on food safety and agriculture associated diseases and how they affect developing countries and disadvantaged populations, as well as ways to prevent or minimize food safety risks, should be investigated [33]. (2) Nutrition aspect: Proper nutrition is essential to human well-being; however, due to the effects of food insecurity, many people are affected by malnutrition, which is a complex, multisectoral issue that ranges from the double burden of malnutrition (where both undernutrition and overnutrition exists in the same society) to non-communicable diseases. The nutrition aspect also deals with evaluating policies and programs that aim to improve the diets, nutritional status, and health of people through critical stages of the lifecycle [33]. (3) Agricultural aspect: The key to reducing poverty and improving food security issues worldwide is to focus on the development of innovative food products using highly nutritious raw ingredients that can provide adequate nutrition for majority of people who are disadvantaged and living in developing countries [33]. Topics of discussion could include fortification, biofortification (process of improving the nutritional quality of food crops using modern biotechnology), the lack of nutrients or vitamins seen in people in countries where food insecurity is prevalent, ways to improve nutrition and vitamin and mineral intake, and the effects of lack of nutrition during critical stages of the lifecycle. Several government policies have been initiated to address the issue of food insecurity in South Africa, such as the food fortification program, food supplementation, and school feeding programs, including the National School Nutrition Program, as well as daycare center schemes [4].

It is noted that National School Nutrition Programs aim to foster a better quality of education by enhancing children’s active learning capacity, alleviating short-term hunger, providing a positive incentive for learners to attend school regularly and punctually, and lastly to address micronutrient deficiencies [4]. Early childhood is an important period of growth and development, both physically and cognitively, and thus requires an optimal dietary intake of energy and nutrients [34]. Nutrient-dense snacks are important to optimize children’s nutritional status and cognitive development, as well as to promote physical growth [35]. According to Greenhalgh, Kristjansson [36], the reasons why school feeding programs do not work could stem from a number of factors, such as the food provided does not provide adequate amounts of the missing nutrients; the type of ingredients used may have a low bioavailability, making it difficult for the body to absorb the necessary nutrients; supplementation occurs too late, whereby older children are far to malnourished to be able to reverse the effects of malnutrition by means of a single meal in the day, which supports the notion that targeting the younger school children would greatly improve the success of the school feeding program. Factors that may improve the efficacy of school feeding programs include designing a school feeding program specifically for the needs of that particular community using local teams rather than distance experts, making sure food is developed to confirm palatability and acceptability, and having measures in place to ensure that food is actually being consumed, such as close supervision during feeding times [14,36].

In this review, several modalities have been used in the product development of snack foods for school feeding programs in Africa: staple foods, food fortification, evaluation of nutritional outcomes, sensory acceptability, and cost-efficacy. Given the central role of staple crops in human nutrition, agricultural production, and food security at large, staple foods are often used in snack product development in resource-constrained settings [37]. Being locally produced foods, their inexpensive nature and energy-dense nutritional offering make them suitable ingredient choices in snack food development for school feeding programs [38]. Food fortification is another modality that has been used to improve the nutritional status of populations. With its cost-effectiveness, food fortification has the potential to significantly benefit the nutritional wellbeing of large segments of populations [39]. Most snack product development initiatives were aligned to nutritional outcomes, with significant benefits; however, adoption and rollover of these snack foods into the mainstream school feeding programs were not evident. In general, the appraisal of the review highlights certain missing components of the product development process of snack foods for school feeding programs in terms of product success at scale, particularly ideation, consumer acceptability, and cost-efficacy.

Certain limitations of this scoping review were identified. Specific databases, such as Scopus, were not used as main sources of information but as secondary sources through Google Scholar. Hence, the review may have missed some relevant studies, and searching other databases may have identified additional pertinent studies. Screening of articles was done by a single reviewer, increasing the margin for error; however, extraction of articles was done by two reviewers. The appraisal process of the review did not assess the quality of studies. Instead, this was guided by the conformance to steps of new food product development for long-life products, a measure that is used mainly in the food industry and often excludes the measurement of health outcomes. This review only included studies in Africa. Snack development for school feeding programs in other developing countries outside Africa would have added more value to the study.

## 5. Conclusions

There is a global consensus that school feeding programs generate a lasting impact that can shape the future of a nation. Upon review of previous snack development initiatives for school feeding programs in Africa, key points have been identified, which have the potential to inform future snack development for school feeding programs. The impact and positive effects of nutrient- and energy-dense snacks on the growth and development of school learners confirm the importance of various authors’ publications. It is recommended that snack development for school feeding programs be grounded in a hybrid model of food product development processes with health outcomes, taking into account children’s nutritional needs in specific countries to ensure maximum impact on learning outcomes and sustainability for mass production.

## Figures and Tables

**Table 1:** Review of previous snack development studies.

Author, Year, Location	Study Design	Duration, Number of Participants, Age of Study Population	Intervention: Product Developed (Ingredients)	Outcomes and Classification of Outcome	Results of Study	Appraisal	Limitations
Ash, Tatala [24] (2003) Mpwapwa district, Tanzania	Randomized, double-blind, placebo-controlled trial	Duration: 6 months; 841 rural primary school children aged 6–11 years	Multiple-micronutrient beverage powder fortified with ten micronutrients developed and produced by food technologists at Procter and Gamble.	Effect of a micronutrient-fortified beverage on iron status, anemia, vitamin A status, and growth of the schoolchildren in Tanzania. Classification: Nutritional	Non-significant differences at BL between children in the fortified and non-fortified groups for iron status and serum retinol. Improved anthropometric measurements in the fortified group (mean incremental changes in weight (1.79 compared to 1.24 kg), height (3.2 compared to 2.6 cm), and BMI (0.88 compared to 0.53). The prevalence of low serum retinol levels (<200 µg/I) dropped from 21.4% to 11.3% in the fortified group.	Steps of product development: Ideation and screening of ideas not indicated. No consumer trials or test marketing. Food Safety: NI Packaging: Sachet Storage: NI Transport: NI	Eleven students were excluded from the entire study due to them having severe anemia. The amounts of the nutrients selected for the fortified beverage were estimates because no previous efficacy data existed for a beverage fortified with multiple micronutrients. Sensory acceptability tests not indicated. No cost analysis.
van Jaarsveld, Faber [25] (2005) Durban, Kwa Zulu Natal, South Africa	Randomized, controlled, unmasked, feeding trial	Duration: 53 school days; 180 semi-rural primary school children aged 5–10 years	Cooked orange-flesh sweet potato and white-flesh sweet potato as the placebo.	The effect of daily consumption of boiled and mashed orange-fleshed sweet potato on vitamin A status of primary school children. Classification: Nutritional	The proportion of children with low serum retinol concentration (<0.070 µmol/L) after intervention decreased from 71% to 50% (*p* = 0.001) in the treatment group and decreased from 73% to 49% (*p* = 0.001) in the control group.	Steps of product development: Ideation and screening of ideas not indicated. No consumer trials or test marketing. Food Safety: Class monitors observed cooking and serving. Packaging: n/a Storage: served immediately Transport: n/a	The study did not assess the prevalence or degree of helminthic infections. No intervention took place during the school holidays. No sensory acceptability tests. No cost analysis. The availability of orange flesh sweet potato was dependent on seasonality.
Murphy, Gewa [26] (2007) Kenya	Randomized, controlled design	Duration: 2 years; 900 school children aged 7–9 years	Three equicaloric snacks were developed: a vegetarian snack designated the “energy snack,” a snack that included beef, and a snack that included whole milk. All snacks were designed to include the local staple food, githeri (a stew of maize, beans, and vegetables). The energy snack consisted of 230 g of vegetarian githeri, the meat snack contained 225 g of githeri with 38% cooked minced beef. To include milk in a snack, the amount of vegetarian githeri was reduced to 100 g, and a glass of milk (250 g) was included in the snack.	Effect of snack design on micronutrient deficiencies in rural Kenyan schoolchildren. Classification: Nutritional	Significant increase in energy intake and Vitamin B12 for animal-based snack. Control group increased energy intakes by 18 kJ/d. Meat group increased energy intakes by 536 kJ/d.	Steps of product development: Ideation and screening of ideas not indicated. Food Safety: NI Packaging: n/a Storage: n/a Transport: n/a	Home intake and intake from the study were not evaluated side-by-side. Breaks in feeding due to weekends, school holidays, or days missed due to illness. No cost analysis.
van Stuijvenberg, Dhansay [27] (2007) South Africa	Cross-sectional study	Duration: 2.5 years; 115 primary school children aged 6–11 years	Shortbread-based biscuit fortified with Β-carotene, iron; and iodine-fortified biscuit, served with a cold drink fortified with vitamin C.	Effect of a micronutrient-fortified biscuit on micronutrient deficiencies in primary school children. Classification: Nutritional	The micronutrient-fortified biscuit was enough to maintain serum retinol concentrations on a day-to-day basis, however, was not enough to sustain levels during the long school holiday. Prevalence of low urinary iodine dropped from 97.1% before the intervention to 4.8% after the first 12 months of intervention.	Steps of product development: Ideation and screening of ideas not indicated. No consumer trials or test marketing. Food Safety: NI Packaging: NI Storage: NI Transport: NI	No intervention took place during the school holiday. Logistical issues prevented the delivery of a vitamin C-fortified cold drink for nine months. The biscuit was baked at 210 °C, which may have possibly degraded the iron amino acid chelate used in the biscuit. No sensory acceptability tests. No cost analysis.
Zivkovich [28] (2011) Roche Village, Rorya District, Tanzania, East Africa	Non-randomized, experimental design	Duration: 15 months; 363 primary school children aged 4.5–11 years	Uji (Corn based porridge made from the root of the cassava plant and millet grain).	The impact of a 130-kcal supplemental snack on growth of primary school children aged 4½ to 11 years old. Classification: Nutritional	Significant decline in the mean height-for-age z-scores (−0.37952) and mean weight-for-age z-scores (−0.19452) from BL to F/U. (BL = 2.2% underweight, 5.3% stunted, and 0.9% wasted; F/U = no student had a z-score of <−2 SD for underweight, stunting, or wasting.	Steps of product development: Ideation and screening of ideas not indicated. No consumer trials or test marketing. Food Safety: NI Packaging: n/a Storage: n/a Transport: n/a	No control group (study was a pre–post study design). Reduced reliability of anthropometric measures due to learners taking the measurements for themselves. No sensory acceptability tests. No delivery of ingredients on certain days. Variation in the preparation methods. No cost analysis.
du Plessis [29] (2010) South Africa	Cross-sectional study	Duration: Not indicated; 209 children aged 9–13 years	Biscuit enhanced with soy flour (nutty wheat flour, Supro Max 6010, flavored sprinkle, baking powder, margarine, egg, fat-free milk).	Development of a nutritious, acceptable, and affordable snack food to prevent obesity in children. Classification: Nutritional, Sensory, Cost effectiveness	An affordable, safe, and acceptable food product with a low-fat and high-density nutritional profile, with at least 20% of RDI for protein and iron was successfully developed. Sensory evaluation: 58.3% liked the taste, 57% liked the texture, 54.3% liked the color, 59.5% liked the smell, and 48.9% liked the portion size. Cost per 30 g portion of the biscuit was priced at 0.55 ZAR (~0.031 USD).	Steps of product development: Ideation and screening of ideas not indicated. No test marketing. Food Safety: Microbiological analysis conducted Packaging: NI Storage: NI Transport: NI	The final snack item was not sent for analysis again. This study was only carried out in 2 primary schools. Soy flour (Supro Max 60,100) is not readily available to consumers.
Kearney, Oldewage–Theron [30] (2011) South Africa	Experimental product development study	Duration: 6 months; 580 rural children aged 6–13 years	A nutritious vetkoek (bread type cake fried in oil).	The development of a nutritious novel food product that is acceptable to children, and rich in energy, protein, and micronutrients. The product should be affordable, cost-effective, and easy to prepare using locally available raw materials with minimal waste and no need for specific fortification or enrichment. Classification: Nutritional, sensory, cost-effectiveness	Sensory acceptability tests showed 90% of children found the product to be acceptable, while 65% liked the vetkoek very much. The cost of the product was R1.50 (~0.080 USD) per day for a 120 g portion and contributed to nutrient intakes of 21.6% for energy, 14.4% for calcium, 141% for iron, 62.42% for zinc, and 17.75% for vitamin A. The vetkoek had a shelf life of two days when stored at room temperature (25 °C).	Steps of product development: Ideation and screening of ideas not indicated. Food Safety: Training provided in preparation of the vetkoek. Packaging: n/a Storage: n/a Transport: n/a	More research is needed to test compliance of consumption over a longer period (at least 12 months). No market needs analysis.
Hochfeld, Graham [31] (2016) Johannesburg, South Africa	Pre- and post-test design	Duration: 10 months; 6656 learners aged 6–17 years received the intervention. 857 learners were used for anthropometric sampling	Fortified cooked porridge (oats, maize, wheat, and sorghum).	Effect of an in-school breakfast program on the anthropometric and school performance of school children. Classification: Nutritional, school performance	There was a positive and statistically significant nutritional change over the period of the program. Reductions in the numbers of overweight and stunted children were seen (BL = 27.6% of learners were either overweight (1.9%) or severely overweight (10.7%). F/U = Overweight (13.8%) and severely overweight (6.4%)). Learners, educators, and principals indicated that they believed the breakfast program had a positive impact on the children’s ability to learn by improving their participation and concentration in the classroom.	Steps of product development: Ideation and screening of ideas not indicated. No consumer trials or test marketing. Food Safety: NI Packaging: n/a Storage: n/a Transport: n/a	The inability to conduct an experimental design where one can systematically control for other intervening factors. The design did not control for other factors to some extent, and therefore the nutritional and performance changes cannot be scientifically attributed to the breakfast program. The nutrition program was launched before the baseline data could be collected. School performance data for the first two terms were missing, therefore the data analysis was compromised. No sensory acceptability tests. No cost analysis.
Belayneh, Yetneberk [32] (2018) Ethiopia	Mixed methods: workshop, sensory testing, focus group discussion	Duration: Not indicated; 95 adolescent girls with an average age of 14.3 years	Kinchie (Porridge) made from QPM (quality protein maize) coarsely milled maize gain.	Stakeholders’ consultation, sensory evaluation, and potential impact of quality protein maize (QPM) for school feeding in Ethiopia Classification: Sensory	QPM kinchie was liked very much by 58% of the participants, whereas only 1.7% rated conventional maize kinchie in this category. None of the participants scored QPM kinchie as poor in terms of taste, however 0.8% of participants scored conventional maize kinchie as poor for taste; 61% of the participants perceived QPM kinchie as very good, whereas only 5% of the respondents perceived conventional maize as very good; 66% of participants rated QPM kinchie to have good texture and 21% (very good texture) and 58% reported conventional maize to have a fair overall sensory characteristic.	Steps of product development: Ideation and screening of ideas not indicated. Food Safety: NI Packaging: n/a Storage: n/a Transport: n/a	Lack of the QPM seed was identified as a possible limitation in the way forward when implementing QPM into school feeding programs. Samples that were served later showed lower score ratings than samples that were served first. No cost analysis.

BL = Baseline; F/U = Follow-up; NI = Not indicated; n/a = Not applicable; QPM = Quality protein maize; RDI = Recommended daily intake; BMI = body mass index.

## References

[1] FAO, IFAD, WFP (2014). The State of Food Insecurity in the World 2014: Strengtherning and Enabling Environment for Food Security and Nutrition.

[2] Bundy D., Burbano C., Grosh M.E., Gelli A., Juke M., Lesley D. (2009). Rethinking School Feeding: Social Safety Nets, Child Development, and the Education Sector.

[3] Buhl A. (2010). Meeting Nutritional Needs through School Feeding: A Snapshot of Four African Nations.

[4] Labadarios D., McHiza Z.J., Steyn N.P., Gericke G., Maunder E.M., Davids Y.D., Parker W.A. (2011). Food security in South Africa: A review of national surveys. Bull World Health Organ.

[5] Department of Basic Education (2019). Basic Education Department Budget Vote 2019/20.

[6] Devereux S., Hochfeld T., Karriem A., Mensah C., Morahanye M., Msimango T., Mukubonda A., Naicker S., Nkomo G., Sanders D. (2018). School Feeding in South Africa: What We Know, What We Don’t Know, What We Need to Do.

[7] South African Department of Basic Education National School Nutrition Programme. https://www.education.gov.za/Programmes/NationalSchoolNutritionProgramme.aspx.

[8] Lesley D., Alice W., Donald B. (2016). Global School Feeding Sourcebook: Lessons from 14 Countries.

[9] South African Department of Basic Education (2016). Report on the Implementation Evaluation of the National School Nutrition Programme.

[10] Lotfi M. (1996). Micronutrient Fortification of Foods: Current Practices, Research, and Opportunities.

[11] Darnton-Hill I., Nalubola R. (2002). Fortification strategies to meet micronutrient needs: Successes and failures. Proc. Nutr. Soc..

[12] Kristjansson B., Petticrew M., MacDonald B., Krasevec J., Janzen L., Greenhalgh T., Wells G.A., MacGowan J., Farmer A.P., Shea B. (2007). School feeding for improving the physical and psychosocial health of disadvantaged students. Cochrane Database of Systemic Reviews.

[13] Galloway R. (2009). School feeding, Outcomes and costs. Food Nutr. Bull..

[14] Powell C.A., Walker S.P., Chang S.M., Grantham-McGregor S.M. (1998). Nutrition and education: A randomized trial of the effects of breakfast in rural primary school children. Am. J. Clin. Nutr..

[15] Costa A.I.A., Jongen W. (2006). New insights into consumer-led food product development. Trends Food Sci. Technol..

[16] Frewer L., Van Trijp H.C. (2007). Understanding Consumers of Food Products.

[17] Sucharew H., Macaluso M. (2019). Progress Notes: Methods for Research Evidence Synthesis: The Scoping Review Approach. J. Hosp. Med..

[18] Tricco A.C., Lillie E., Zarin W., O’Brien K.K., Colquhoun H., Levac D., Moher D., Peters M., Horsley T., Weeks L. (2018). PRISMA Extension for Scoping Reviews (PRISMA-ScR): Checklist and Explanation. Ann. Intern. Med..

[19] Rudder A., Ainsworth P., Holgate D. (2001). New food product development: Strategies for success?. Br. Food J..

[20] Fuller G.W. (2016). New Food Product Development: From Concept to Marketplace.

[21] South Africa (1996). South Africa. South African Schools Act, 1996. No.84 of 1996.

[22] Institute of Medicine AMDR Tables 2010. http://nationalacademies.org/hmd/Activities/Nutrition/SummaryDRIs/DRI-Tables.aspx.

[23] Moher D., Liberati A., Tetzlaff J., Altman D., PRISMA Group (2009). Preferred reporting items for systematic reviews and meta-analyses: The PRISMA statement. Phys. Ther..

[24] Ash D.M., Tatala S.R., Frongillo E.A., Ndossi G.D., Latham M.C. (2003). Randomized efficacy trial of a micronutrient-fortified beverage in primary school children in Tanzania. Am. J. Clin. Nutr..

[25] Van Jaarsveld P.J., Faber M., Tanumihardjo S.A., Nestel P., Lombard C., Benadé A.J.S. (2005). β-Carotene–rich orange-fleshed sweet potato improves the vitamin A status of primary school children assessed with the modified-relative-dose-response test. Am. J. Clin. Nutr..

[26] Murphy S.P., Gewa C., Grillenberger M., Bwibo N.O., Neumann C.G. (2007). Designing snacks to address micronutrient deficiencies in rural Kenyan schoolchildren. J. Nutr..

[27] Van Stuijvenberg M., Dhansay M., Smuts C.M., Lombard C., Jogessar V., Benadé A. (2001). Long-term evaluation of a micronutrient-fortified biscuit used for addressing micronutrient deficiencies in primary school children. Public Health Nutr..

[28] du Plessis R.M. (2010). Development of a Nutritious, Acceptable and Affordable Snack Food to Prevent Obesity in Children.

[29] Zivkovich C.J. (2011). An Evaluation of a Supplemental Snack Feeding Program on Growth in School-Aged Children Living in Rural Tanzania, East Africa.

[30] Kearney J., Oldewage–Theron W., Napier C. (2011). Development and processing of a novel food product for a school feeding project in South Africa. Afr. J. Hosp. Tour. Leis..

[31] Hochfeld T., Graham L., Patel L., Moodley J., Ross E. (2016). Does school breakfast make a difference? An evaluation of an in-school breakfast programme in South Africa. Int. J. Educ. Dev..

[32] Belayneh D., Yetneberk S., Teklewold A., Groote H. (2018). Quality protein maize (QPM) for school feeding in Ethiopia: Stakeholders consultation, sensory evaluation and potential impact. J. Nutr. Health Food Eng..

[33] FAO, IFAD, UNICEF, WFP, WHO (2017). The State of Food Security and Nutrition in the World 2017.

[34] Ogata B.N., Hayes D. (2014). Position of the Academy of Nutrition and Dietetics: Nutrition Guidance for Healthy Children Ages 2 to 11 Years. J. Acad. Nutr. Diet..

[35] Shriver L.H., Marriage B.J., Bloch T.D., Spees C.K., Ramsay S.A., Watowicz R.P., Taylor C.A. (2017). Contribution of snacks to dietary intakes of young children in the United States. Matern. Child Nutr..

[36] Greenhalgh T., Kristjansson E., Robinson V. (2007). Realist review to understand the efficacy of school feeding programs. BMJ.

[37] Molinas L., de la Mothe M.R. (2010). The multiple impacts of school feeding: A new approach for reaching sustainability. Revolution: From Food Aid to Food Assistance. Innovations in Overcoming Hunger.

[38] World Food Programme (WFP) Home-Grown School Feeding Resource Framework. https://www.wfp.org/home-grown-school-feeding.

[39] World Health Organization (WHO), Food and Agriculture Organisation of the United Nations (FAO) (2006). Guidelines on Food Fortification with Micronutrients.

